# The role of albumin–globulin ratio in peripheral arterial disease among hypertensive adults: evidence from a large-scale multicenter study

**DOI:** 10.3389/fendo.2026.1839770

**Published:** 2026-05-28

**Authors:** Chao Yu, Yang Yang, Xiong Liao, Lei Wu

**Affiliations:** 1 Department of Epidemiology, School of Public Health, Jiangxi Medical College, Nanchang University, Nanchang, Jiangxi, China; 2 Center for Prevention and Treatment of Cardiovascular Diseases, the Second Affiliated Hospital of Nanchang University, Nanchang, Jiangxi, China; 3 Jiangxi Provincial Key Laboratory of Disease Prevention and Public Health, Nanchang University, Nanchang, Jiangxi, China; 4 Department of Nutrition, The First Affiliated Hospital, Jiangxi Medical College, Nanchang University, Nanchang, Jiangxi, China

**Keywords:** albumin globulin ratio, hypertension, multicenter stugy, peripheral arterial disease, real world study

## Abstract

**Objective:**

With the aging population of China, a higher prevalence of peripheral arterial disease (PAD) is observed. The albumin-globulin ratio (AGR) has a certain value in predicting cardiovascular mortality, but little research has been conducted on the association between AGR and PAD. Therefore, this study aims to explore the association between AGR and PAD in hypertensive adults.

**Methods:**

This retrospective cross-sectional study recruited a total of 28, 684 participants from March 2018 to August 2018 in Wuyuan County, Jiangxi Province, and Yuexi County, Anhui Province. A generalized additive model was utilized to analyze the relationship between AGR and PAD.

**Result:**

A total of 24, 808 hypertensive adults were included in this study. The regression analysis of the generalized additive model revealed an inverse association between AGR and PAD when AGR was set as a continuous variable. Further analysis with AGR divided into quartiles indicated a lower risk of peripheral artery disease in the Q2, Q3, and Q4 groups compared with the Q1 group. Specifically, in the fully adjusted model (Model 3), the risk of peripheral artery disease in the Q2, Q3, and Q4 groups decreased by 11% (OR: 0.89; 95%CI: 0.71-1.10; *P* = 0.278), 27% (OR: 0.73; 95%CI: 0.58-0.92; *P* = 0.008), and 35% (OR: 0.65; 95%CI: 0.51-0.84; *P* < 0.001) compared with the Q1 group, respectively. Subgroup analysis found that the relationship between AGR and PAD was more significant in the smoking and stroke populations.

**Conclusion:**

A negative association was observed between AGR and PAD, with this relationship being particularly pronounced in individuals who smoke or have a history of stroke.

## Graphical Abstract

**Figure:**
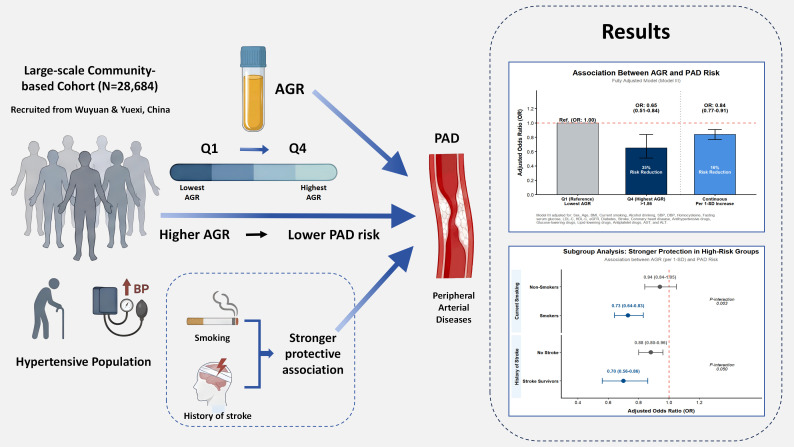

## Introduction

Peripheral arterial disease (PAD) is characterized by peripheral circulatory dysfunction. In a broad sense, PAD refers to atherosclerotic lesions caused by major arteries other than the coronary artery and the aorta. In a more specific context, PAD refers to lower extremity arterial disease. In the present study, the ankle-brachial index was used to define PAD. According to statistics, about 330 million people suffer from cardiovascular diseases in China. Among them, hypertension shows the highest prevalence, with about 245 million people, followed by PAD, with about 45.3 million people (1). With the aging of the population, the incidence and prevalence of cardiovascular diseases such as hypertension, PAD, heart failure, and coronary heart disease are still rising (1). The current global statistics show that 236 million people suffer from PAD (2). The REACH study also revealed that 18% of PAD patients undergo amputation, indicating a high number of patients not receiving timely and effective treatment. The China Hypertension Survey found a prevalence of 6.6% for PAD in the natural population aged ≥ 35 years in China. Moreover, the awareness rate and good prognosis rate of PAD in China are low, with only 4.9% and 0.2%, respectively (3). This disease has an insidious onset, showing no symptoms in the early stage. As the disease progresses, unilateral or bilateral muscle soreness may occur during exercise, resulting in claudication symptoms, which are relieved after a few minutes of rest. Upon further disease progression, lower extremity soreness may occur even at rest, which is referred to as ischemic resting pain, ultimately leading to foot ulcers and amputation. Meanwhile, a higher occurrence of other cardiovascular events and cardiovascular mortality has been reported (4), imposing a serious burden. The risk factors for PAD include hyperlipidemia, smoking, obesity, hypertension, diabetes, renal insufficiency, age, high-sensitivity C-reactive protein, vitamin D deficiency, hyperhomocysteinemia, hyperuricemia, etc. (5–8). However, despite effective control of these traditional and non-traditional risk factors, PAD still cannot be completely prevented. Therefore, further research should be conducted to identify additional hidden risk factors for PAD.

Serum albumin reflects the body’s overall nutritional status and is involved in the transportation of many endogenous and exogenous substances, demonstrating anti-inflammatory (9), antioxidant (10), and anticoagulant physiological properties (11). Elevated serum globulin levels lead to an increase in blood viscosity and are associated with chronic inflammation (12). The albumin-globulin ratio (AGR) is a new predictive indicator. Previous studies have revealed that AGR has a good predictive role in the prognostic evaluation of various cancers, including colorectal cancer (13), nasopharyngeal carcinoma (14), breast cancer (15), lung cancer (16), and pancreatic cancer (17, 18). In addition, previous studies have shown that AGR is related to cardiovascular mortality (19), all-cause mortality (20), and myocardial infarction (21). Based on the physiological characteristics of albumin and globulin, a certain connection may be observed between AGR and PAD. Therefore, this study included nearly 30, 000 hypertensive people in Wuyuan, Jiangxi, and Yuexi, Anhui to explore the relationship between AGR and the risk of PAD in the hypertensive population.

## Subjects and methods

### Study design and population

This study included patients from the China H-Type Hypertension Registry Study (Registration Number: ChiCTR1800017274), which aims to establish a national registry of hypertensive patients to investigate the prevalence and treatment of hypertension in China and evaluate the factors affecting hypertension and its prognosis. Inclusion criteria: (1) Age ≥ 18 years. (2) Diagnosed with hypertension, defined as blood pressure measured on at least three separate days, with at least two measurements meeting the criteria of systolic blood pressure (SBP) ≥ 140 mmHg and/or diastolic blood pressure (DBP) ≥ 90 mmHg, self-reported history of hypertension, or use of antihypertensive drugs within 2 weeks. Blood pressure was measured after the subjects emptied their bladder and sat resting for at least 30 minutes. Each blood pressure measurement was taken at least 1 minute apart, and the average of three measurements was recorded. Exclusion criteria:(1) Subjects with psychological or neurological impairment preventing them from providing informed consent. (2) Subjects who cannot be followed up according to the study protocol or plan to relocate in the near future.(3) Patients who were assessed by the research doctor as ineligible for inclusion or long-term follow-up. This study was approved by the Ethics Committee of the Second Affiliated Hospital of Nanchang University (NO. 2018019) and the Ethics Committee of the Institute of Biomedicine of Anhui Medical University (NO.CH1059). All participants provided signed informed consent.

A total of 28, 684 participants were recruited from March 2018 to August 2018 in Wuyuan, Jiangxi, and Yuexi, Anhui. However, malignant tumors and severe liver damage can significantly affect the AGR, so 443 patients with malignant tumors and 69 patients with severe liver dysfunction, defined as alanine aminotransferase (ALT) or aspartate aminotransferase (AST) levels exceeding 3 times the upper normal limit (22), were excluded. Furthermore, 59 individuals with missing ankle-brachial index and AGR data were excluded. Additionally, 4 outlying observations with total protein ≤ albumin were removed. Subsequently, 3, 277 participants with missing ankle-brachial index and 24 with an ankle-brachial index ≥1.4 were excluded due to potential vascular calcification compromising diagnostic accuracy (23). Finally, 24, 808 subjects were included in the analysis (**Supplementary Figure 1**).

### Baseline data collection

The baseline data of this study originated from questionnaires, physical measurements, and laboratory tests, and were collected by professionally trained clinicians through a standardized investigation protocol. The questionnaire included sociodemographic characteristics (gender, age, etc.), smoking history, drinking history, disease history (stroke, coronary heart disease, heart failure, etc.), and combined medication history (whether to take antihypertensive drugs, hypoglycemic drugs, lipid-lowering drugs, antiplatelet drugs, etc.). Anthropometric indicators included height, weight, blood pressure, pulse, etc.; the height and weight were measured to the nearest 0.5 cm and 0.5 kg, respectively. The participants were informed to come on an empty stomach, take off their shoes, and keep the thinnest clothes. The measuring ruler was fixed vertically to the ground, and the participants were instructed to stand upright, with their feet flat on the ground, heels close to the measuring ruler, and shoulders and hips close to the measuring ruler. Blood pressure was measured after the subjects emptied the bladder for at least 5 minutes and rested for at least 30 minutes. Each measurement was taken at least 1 minute apart, and the average of three measurements was calculated. Laboratory tests included homocysteine, fasting blood glucose, total cholesterol, triglycerides, high-density lipoprotein, blood uric acid, serum creatinine, total protein, albumin, alanine aminotransferase, aspartate aminotransferase, and other biochemical indicators, which were detected by an automatic clinical analyzer (Beckman Coulter).

### Definition of indicators

Body mass index (BMI) was calculated as weight/height squared (kg/m2). The estimated glomerular filtration rate (eGFR) was calculated using the Chronic Kidney Disease Epidemiology Collaboration equation (CKD-EPI). AGR was calculated as [albumin/(total protein-albumin)]. Diabetes was defined as having a history of diabetes, taking hypoglycemic drugs, or having a fasting blood glucose > 7.0 mmol/L during the screening period. Chronic kidney disease was defined as eGFR < 60 mL/min per 1.73 m^2^. Hypertension: was defined as SBP ≥ 140 mmHg and/or DBP ≥ 90 mmHg or taking antihypertensive drugs within 2 weeks or having a history of hypertension. Lipid-lowering drugs included any statin or fibrate drugs.

PAD measurement: Using the Omron Colin BP-203 RPE III device (Omron Health Care), the SBP of each arm and the dorsalis pedis and posterior tibial arteries of each ankle were measured by Doppler ultrasound. PAD was defined as an ankle-brachial index (ABI) <0.9 in either leg (24). All blood pressure measurements for ABI were performed by trained and standardized technicians using a validated oscillometric device. At each center, the measurement was repeated twice for each limb, and the average values were used to calculate the ABI. Regular cross-center calibration and quality control audits were conducted throughout the study period.

### Statistical methods

According to the quartile grouping of AGR, the baseline population was divided into four groups: Q1, Q2, Q3, and Q4. Continuous variables were expressed as mean ± standard deviation and categorical variables were expressed as frequency (%). The chi-square test was employed to analyze categorical variables, and the one-way analysis of variance (ANOVA) was used to compare the continuous variables of the four groups. The generalized additive model was used to evaluate the association between AGR and PAD. A total of four models were constructed to evaluate their relationship. Crude model: no variable adjustment; Model 1: adjusting for gender, age, BMI, smoking, and drinking; Model 2: same adjustments as Model 1, with further adjustments for SBP, DBP, homocysteine, fasting blood glucose, high-density lipoprotein cholesterol, low-density lipoprotein cholesterol, diabetes, stroke, coronary heart disease, antihypertensive drugs, lipid-lowering drugs, antiplatelet drugs, and glomerular filtration rate; Model 3 (fully adjusted) was further adjusted for ALT and AST based on Model 2. The adjusted covariates were based on the confounding factors mentioned in the previous literature. Variables that were significant in the univariate analysis, those with an effect estimate change greater than 10% after adjusting for confounders, and those deemed clinically important were included in the final model. In addition, the generalized additive model and the smooth fitting curve (penalized spline method) were utilized to describe the dose-response relationship between AGR and PAD. Moreover, sensitivity analysis was performed to evaluate whether there were effect modifiers that could change the association between AGR and PAD in subgroups based on gender (male vs. female), age (< 65 vs. ≥ 65 years), BMI (< 18.5 vs. ≥ 18.5, < 24 vs. ≥ 24 kg/m^2^), smoking, drinking, chronic kidney disease, diabetes, serum total cholesterol (≥ 5.2 vs. < 5.2 mmol/L), homocysteine (< 15 vs. ≥ 15 mmol/L), stroke, coronary heart disease, antihypertensive drugs, lipid-lowering drugs, antiplatelet drugs, and low-density lipoprotein cholesterol (<= 3.1 vs. > 3.1 mmol/l). Joint analysis was used to further explore the relationship between AGR and PAD. To address the issue of multiple hypothesis testing, a Bonferroni correction was applied to the interaction tests across the 14 examined subgroups, setting the significance threshold at 0.05/14 ≈ 0.0036. The uncorrected results are also presented to facilitate exploratory data interpretation. All subgroup analyses should be considered hypothesis-generating. As a *post-hoc* sensitivity analysis, the Q1 and Q2 groups were combined and the analysis was repeated; these results are presented in the **Supplementary Material** (**Supplementary Table 1**). All data analysis in this study was performed using Empower (R; www.empowerstats.net/; X&Y Solutions, Inc, Boston, MA) and the statistical software package (R) (http://www.R-project.org, The R Foundation 3.5.1). In this study, a two-sided P-value of less than 5% was considered statistically significant.

## Results

### Baseline characteristics of the study population

**Table 1** presents the baseline characteristics of the study population according to AGR quartiles. The average age of this population was 60.8 ± 9.6 years, including 11, 717 males, representing 47.2% of the cohort. Specifically, individuals with a higher AGR exhibited a lower average age, serum total protein level, low-density lipoprotein cholesterol level, uric acid level, aspartate aminotransferase level, and the prevalence of stroke, PAD, and diabetes. Moreover, the group showed a higher proportion of males, glomerular filtration rate, serum albumin level, and smoking and drinking rates. No statistically significant difference in the use of antiplatelet drugs, lipid-lowering drugs, and antihypertensive drugs was found among the groups.

**Table 1:** Characteristics of study participants by AGR.

Variables	Total	AGR quartile				*P*-value
		Q1 (<1.56)	Q2 (1.56-<1.70)	Q3 (1.70-<1.86)	Q4 (≥1.86)	
N	24808	6172	6223	6203	6210	
Male, n (%)	11717 (47.2)	2233 (36.2)	2649 (42.6)	3042 (49.0)	3793 (61.1)	<0.001
Age, year	60.8 ± 9.6	62.2 ± 9.8	60.9 ± 9.6	60.5 ± 9.4	59.5 ± 9.3	<0.001
BMI, kg/m^2^	24.1 ± 3.7	24.2 ± 4.1	24.2 ± 3.6	24.2 ± 3.5	24.0 ± 3.5	0.003
SBP, mmHg	146.7 ± 18.1	147.4 ± 18.6	146.8 ± 18.1	146.6 ± 18.1	145.8 ± 17.7	<0.001
DBP, mmHg	91.0 ± 11.0	90.1 ± 11.2	90.9 ± 10.9	91.3 ± 10.9	91.6 ± 10.8	<0.001
Current smoking, n (%)	6923 (27.9)	1302 (21.1)	1511 (24.3)	1763 (28.4)	2347 (37.8)	<0.001
Current alcohol drinking, n (%)	5391 (21.7)	1003 (16.3)	1226 (19.7)	1432 (23.1)	1730 (27.9)	<0.001
Homocysteine, µmol/L	16.6 ± 10.5	17.2 ± 10.8	16.4 ± 10.2	16.3 ± 9.9	16.6 ± 10.9	<0.001
Fasting serum glucose, mmol/L	6.1 ± 1.5	6.2 ± 1.8	6.1 ± 1.6	6.0 ± 1.4	5.9 ± 1.3	<0.001
Total cholesterol, mmol/L	5.2 ± 1.1	5.4 ± 1.2	5.3 ± 1.1	5.2 ± 1.1	5.0 ± 1.0	<0.001
AST, U/L	24.2 ± 9.4	25.7 ± 10.5	24.3 ± 9.2	24.0 ± 9.1	23.0 ± 8.3	<0.001
ALT, U/L	18.5 ± 11.6	18.8 ± 12.2	18.4 ± 11.3	18.6 ± 11.6	18.4 ± 11.3	0.171
Uric acid, µmol/L	403.4 ± 116.5	417.1 ± 123.1	404.0 ± 117.6	397.1 ± 114.0	395.4 ± 109.9	<0.001
Serum total protein, g/L	74.0 ± 6.3	77.1 ± 6.7	75.0 ± 5.8	73.4 ± 5.5	70.5 ± 5.3	<0.001
Serum albumin, g/L	46.5 ± 3.7	45.3 ± 4.0	46.5 ± 3.6	47.0 ± 3.5	47.2 ± 3.4	<0.001
AGR	1.7 ± 0.2	1.4 ± 0.1	1.6 ± 0.0	1.8 ± 0.0	2.0 ± 0.2	<0.001
HDL, mmol/L	1.6 ± 0.4	1.6 ± 0.4	1.6 ± 0.4	1.6 ± 0.4	1.5 ± 0.4	<0.001
LDL, mmol/L	3.1 ± 0.8	3.2 ± 0.9	3.1 ± 0.8	3.1 ± 0.8	2.9 ± 0.7	<0.001
eGFR, ml/min/1.73 m^2^	91.0 ± 18.4	87.3 ± 20.9	90.5 ± 18.4	92.3 ± 17.1	93.9 ± 16.3	<0.001
Diabetes, n (%)	4022 (16.2)	1200 (19.4)	1010 (16.2)	951 (15.3)	861 (13.9)	<0.001
Coronary heart disease, n (%)	2427 (9.8)	618 (10.0)	636 (10.2)	613 (9.9)	560 (9.0)	0.116
Chronic kidney disease, n (%)	1486 (5.99%)	431 (6.98%)	370 (5.95%)	357 (5.76%)	328 (5.28%)	<0.001
Stroke, n (%)	3110 (12.54%)	827 (13.40%)	814 (13.08%)	744 (11.99%)	725 (11.67%)	0.008
PAD, n (%)	595 (2.4)	202 (3.3)	159 (2.6)	126 (2.0)	108 (1.7)	<0.001
Antihypertensive drugs, n (%)	16334 (65.84)	4076 (66.04)	4118 (66.18)	4122 (66.45)	4018 (64.70)	0.168
Glucose-lowering drugs, n (%)	975 (3.9)	280 (4.5)	233 (3.7)	221 (3.6)	241 (3.9)	0.032
Lipid-lowering drugs, n (%)	748 (3.0)	163 (2.6)	186 (3.0)	205 (3.3)	194 (3.1)	0.172
Antiplatelet drugs, n (%)	1102 (4.4)	264 (4.3)	288 (4.6)	253 (4.1)	297 (4.8)	0.210

Data are expressed as mean ± SD, numbers (percentage) or Quartile (IQR) as appropriate. PAD, peripheral arterial disease; OR, odds ratio; 95% CI, 95% confidence interval; AGR, albumin globulin ratio; BMI, body mass index (Calculated as weight in kilograms divided by height in meters squared); SBP, systolic blood pressure; DBP, diastolic blood pressure; AST, Aspartate Aminotransferase; ALT, alanine aminotransferase; HDL-C, high-density lipoprotein cholesterol; LDL-C, low-density lipoprotein cholesterol; eGFR, estimated glomerular filtration rate.

### Association of albumin-globulin ratio and peripheral arterial disease

**Figure 1:**
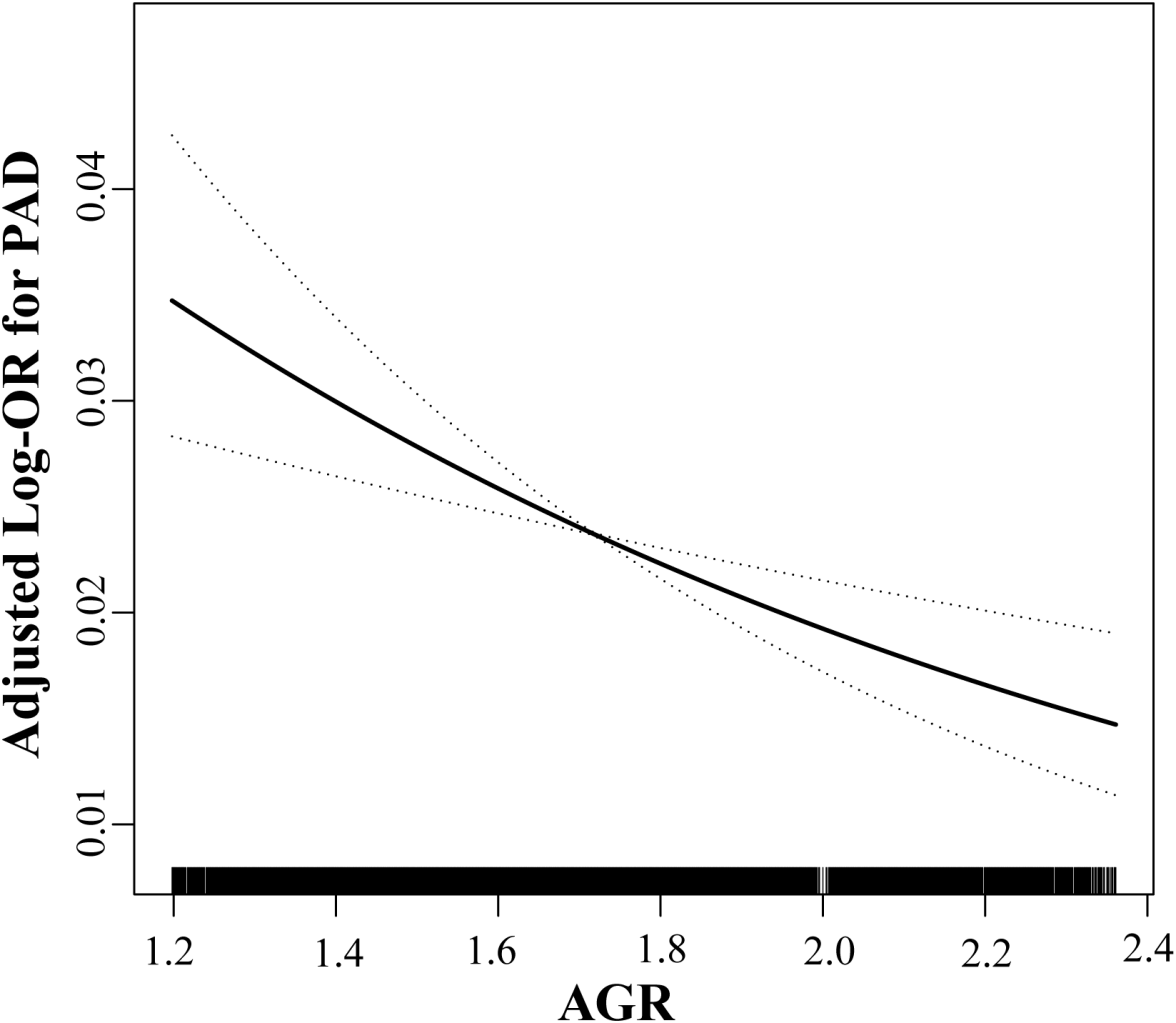
The smoothed curve depicts the association between the albumin-globulin ratio and the risk of peripheral artery disease in hypertensive Chinese patients. A linear correlation was found between AGR and the risk of PAD (P < 0.05). The solid and dashed lines represent the estimated values and corresponding 95% confidence interval, respectively. Model was adjusted for sex, age, BMI, current smoking, current alcohol drinking, SBP, DBP, homocysteine, fasting serum glucose, LDL-C, HDL-C, eGFR, diabetes, stroke, coronary heart disease, antihypertensive drugs, glucose-lowering drugs, lipid-lowering drugs, antiplatelet drugs, AST and ALT. PAD, peripheral arterial disease; OR, odds ratio; 95% CI, 95% confidence interval; AGR, albumin globulin ratio; BMI, body mass index (Calculated as weight in kilograms divided by height in meters squared); SBP, systolic blood pressure; DBP, diastolic blood pressure; AST, Aspartate Aminotransferase; ALT, alanine aminotransferase; HDL-C, high-density lipoprotein cholesterol; LDL-C, low-density lipoprotein cholesterol; eGFR, estimated glomerular filtration rate.

As shown in **Table 2**, AGR was treated as a continuous variable, and each standard deviation increase in AGR was associated with a 16% reduction in the risk of PAD (OR: 0.84; 95% CI: 0.77–0.91) in the fully adjusted model, showing statistical significance (P < 0.05). AGR quartiles were used as a categorical variable and a gradual decrease in PAD risk was observed across models when compared to the Q1 group. In the fully adjusted model, PAD risk decreased by 11% (OR: 0.89; 95% CI: 0.71-1.10) in Q2, 27% (OR: 0.73; 95% CI: 0.58-0.92) in Q3, and 35% (OR: 0.65; 95% CI: 0.51-0.84) in Q4. Since the reduction in PAD risk for Q2 (11%) demonstrated no statistical significance (P = 0.278), the Q1 and Q2 groups were combined, and the analysis was repeated. The results revealed a similar trend, with PAD risk decreasing by 23% (OR: 0.77; 95% CI: 0.63-0.95) in Q3 and 31% (OR: 0.69; 95% CI: 0.55-0.87) in Q4 compared to the Q1–2 group. Subsequently, a smooth curve was used to illustrate the relationship between PAD and AGR (**Figure 1**), indicating a negative correlation between them. As the AGR level increased, the risk of PAD gradually decreased, which was consistent with the regression results.

**Table 2:** Association of AGR with PAD in hypertensive adults.

Variables	Events/N	PAD OR (95% CI), *p* value			
		Crude model	Model 1	Model 2	Model 3
Per SD increment	595/24808	0.76 (0.70, 0.83), <0.001	0.80 (0.73, 0.87), <0.001	0.84 (0.77, 0.92), <0.001	0.84 (0.77, 0.91) <0.0001
AGR quartile					
Q1 (<1.56)	202/6172	Ref.	Ref.	Ref.	Ref.
Q2(1.56, <1.70)	159/6223	0.77 (0.63, 0.96), 0.018	0.84 (0.68, 1.04), 0.102	0.90 (0.72, 1.11), 0.326	0.89 (0.71, 1.10), 0.278
Q3(1.70, <1.86)	126/6203	0.61 (0.49, 0.77), <0.001	0.67 (0.53, 0.84), 0.001	0.74 (0.59, 0.94), 0.012	0.73 (0.58, 0.92), 0.008
Q4 (≥1.86)	108/6210	0.52 (0.41, 0.66), <0.001	0.58 (0.45, 0.74), <0.001	0.67 (0.52, 0.86), 0.002	0.65 (0.51, 0.84), <0.001
*P* for trend		<0.001	<0.001	0.004	<0.001

Model 1 was adjusted for sex, age, BMI, current smoking and current alcohol drinking.

Model 2 was adjusted for Model 1 plus SBP, DBP, homocysteine, fasting serum glucose, LDL-C, HDL-C, eGFR, diabetes, stroke, coronary heart disease, antihypertensive drugs, glucose-lowering drugs, lipid-lowering drugs and antiplatelet drugs.

Model 3 (fully adjusted) was adjusted for Model 2 plus AST and ALT.PAD, peripheral arterial disease; OR, odds ratio; 95% CI, 95% confidence interval; AGR, albumin globulin ratio; BMI, body mass index (Calculated as weight in kilograms divided by height in meters squared); SBP, systolic blood pressure; DBP, diastolic blood pressure; AST, Aspartate Aminotransferase; ALT, alanine aminotransferase; HDL-C, high-density lipoprotein cholesterol; LDL-C, low-density lipoprotein cholesterol; eGFR, estimated glomerular filtration rate.

### Subgroup analysis

**Figure 2:**
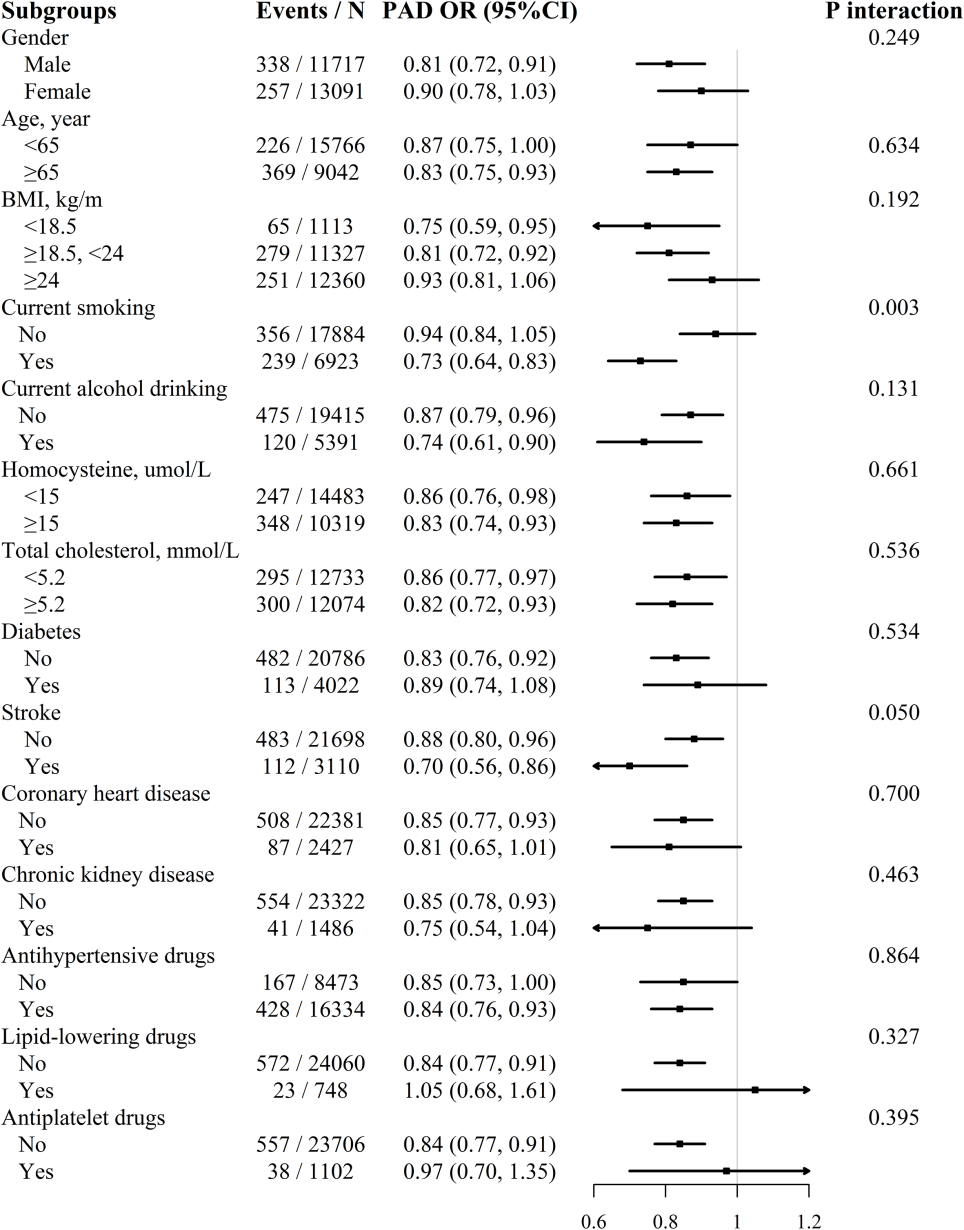
The association between AGR with the risk of PAD in various subgroups. Model was adjusted, if not stratified, for sex, age, BMI, current smoking, current alcohol drinking, SBP, DBP, homocysteine, fasting serum glucose, LDL-C, HDL-C, eGFR, diabetes, stroke, coronary heart disease, antihypertensive drugs, glucose-lowering drugs, lipid-lowering drugs and antiplatelet drugs. PAD, peripheral arterial disease; OR, odds ratio; 95% CI, 95% confidence interval; AGR, albumin globulin ratio; BMI, body mass index (Calculated as weight in kilograms divided by height in meters squared); SBP, systolic blood pressure; DBP, diastolic blood pressure; AST, Aspartate Aminotransferase; ALT, alanine aminotransferase; HDL-C, high-density lipoprotein cholesterol; LDL-C, low-density lipoprotein cholesterol; eGFR, estimated glomerular filtration rate.

The stability of the inverse association between AGR and PAD across different populations and potential effect modifiers was further explored, and a subgroup analysis and interaction test were performed. The results (**Figure 2**) showed that, in general, a higher AGR was associated with a lower risk of PAD in each subgroup, with increasing AGR levels associated with a gradual decrease in PAD risk. Notably, smoking and stroke appeared to influence the relationship between AGR and peripheral arteries. Specifically, in smokers and non-smokers, each standard deviation increase in AGR reduced PAD risk by 27% (OR: 0.73; 95% CI: 0.64-0.83) and 6% (OR: 0.94; 95% CI: 0.84-1.05), respectively (*P* for interaction = 0.003). A borderline significant interaction was observed for stroke (*P* for interaction = 0.050), suggesting a potentially stronger inverse relationship in individuals with a history of stroke. However, this finding should be interpreted with caution given the marginal significance and exploratory nature of subgroup analyses.

### Joint effect

**Figure 3:**
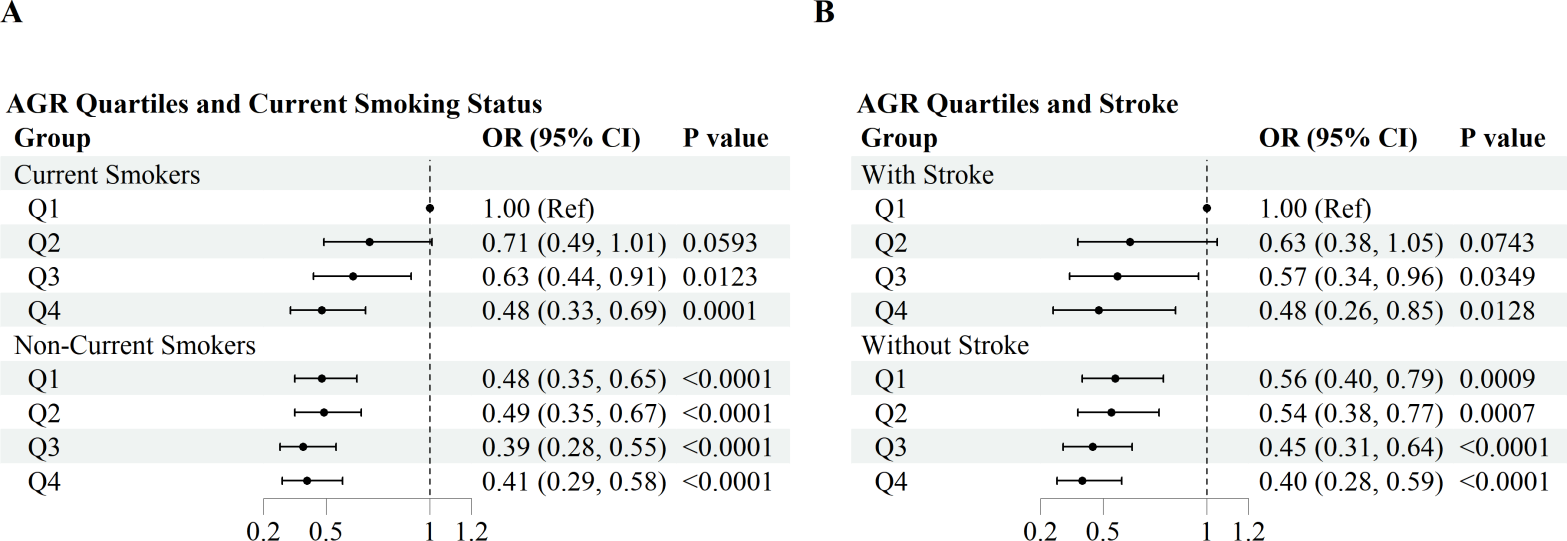
Combined effects of AGR and current smoking **(A)**, AGR and stroke **(B)** on the risk of PAD. Forest plots displaying the odds ratios and 95% confidence intervals for PAD risk by AGR categories, stratified by smoking status and stroke history. Model was adjusted for sex, age, BMI, current smoking, current alcohol drinking, SBP, DBP, homocysteine, fasting serum glucose, LDL-C, HDL-C, eGFR, diabetes, stroke, coronary heart disease, antihypertensive drugs, glucose-lowering drugs, lipid-lowering drugs, antiplatelet drugs, AST and ALT. PAD, peripheral arterial disease; OR, odds ratio; 95% CI, 95% confidence interval; AGR, albumin globulin ratio; BMI, body mass index (Calculated as weight in kilograms divided by height in meters squared); SBP, systolic blood pressure; DBP, diastolic blood pressure; AST, Aspartate Aminotransferase; ALT, alanine aminotransferase; HDL-C, high-density lipoprotein cholesterol; LDL-C, low-density lipoprotein cholesterol; eGFR, estimated glomerular filtration rate.

**Figure 4:**
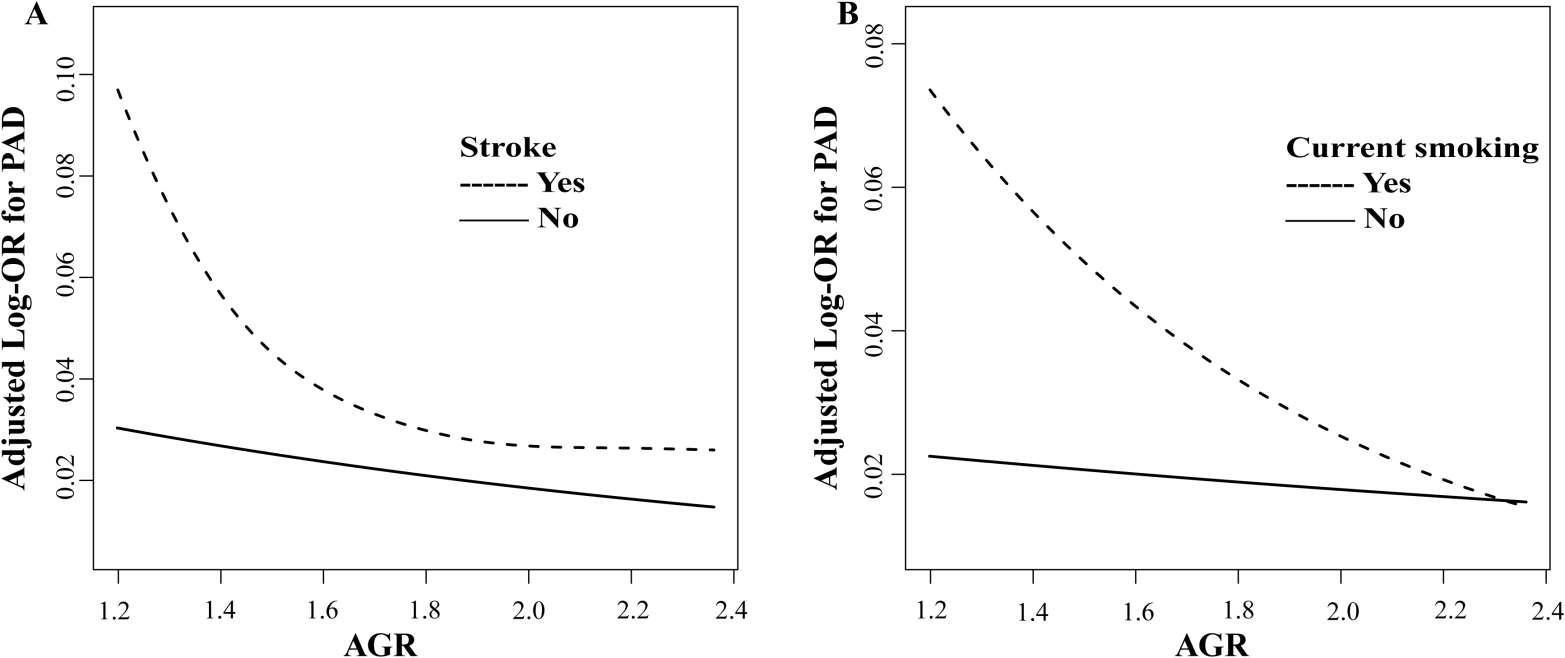
Association between AGR and PAD stratified by stroke **(A)** and current smoking **(B)** Model was adjusted for sex, age, BMI, current smoking, current alcohol drinking, SBP, DBP, homocysteine, fasting serum glucose, LDL-C, HDL-C, eGFR, diabetes, stroke, coronary heart disease, antihypertensive drugs, glucose-lowering drugs, lipid-lowering drugs, antiplatelet drugs, AST and ALT. PAD, peripheral arterial disease; OR, odds ratio; 95% CI, 95% confidence interval; AGR, albumin globulin ratio; BMI, body mass index (Calculated as weight in kilograms divided by height in meters squared); SBP, systolic blood pressure; DBP, diastolic blood pressure; AST, Aspartate Aminotransferase; ALT, alanine aminotransferase; HDL-C, high-density lipoprotein cholesterol; LDL-C, low-density lipoprotein cholesterol; eGFR, estimated glomerular filtration rate.

To further investigate the roles of smoking and stroke in the relationship between AGR and peripheral arterial disease (PAD), a joint effect analysis was performed, stratified by smoking status and stroke history. The results are presented as forest plots in **Figure 3**, showing OR and 95% CI for PAD risk by AGR categories stratified by smoking status and stroke history. A smooth curve analysis was also used to describe the relationship between AGR and PAD in both smoking and non-smoking populations, as well as in stroke and non-stroke populations. The findings demonstrated that the overall risk of PAD was lower in non-smokers and non-stroke individuals, and the inverse relationship between AGR and PAD was more pronounced among smokers and stroke survivors (**Figure 4**).

## Discussion

To the best of our knowledge, this study is the first to explore the relationship between AGR and PAD in the hypertensive population. An inverse association was found between AGR and PAD, revealing a gradually decreasing PAD risk as AGR increases. Subgroup analysis indicated that this result was stable and similar in most subgroups. Particularly, the inverse association between AGR and PAD was more pronounced in smokers and showed a borderline significant interaction in the stroke population. A joint analysis was conducted to further explore these relationships.

Only a few previous studies have explored the relationship between the AGR and PAD. The current study aims to explore the relationship between the AGR and PAD. Yao et al. (25) conducted a retrospective cross-sectional study, which included a total of 266 elderly diabetic patients from the Department of Endocrinology of Nanfang Hospital of Southern Medical University from January 21, 2019, to August 4, 2021. The results showed that the AGR of the high ankle-brachial index group was 1.17 times that of the low ankle-brachial index group (OR: 1.20; 95%CI: 1.05-1.40 vs. OR: 1.40; 95%CI: 1.30-1.50), showing a statistically significant difference. These findings suggested a negative correlation between the AGR and PAD, which is consistent with the results of this study.

Nonetheless, the pathological mechanism of PAD remains incompletely understood. At present, the pathological mechanism of PAD is mainly considered to be related to atherosclerosis, thrombosis, and microvascular dysfunction (26). Multiple studies have investigated the relationship between hypertension and PAD (27–29). Hypertension can damage the vascular endothelium and accelerate the process of atherosclerosis, leading to vascular stenosis and PAD. Some studies have shown that PAD is related to inflammation. Tzoulaki et al. recruited a total of 1, 519 natural people aged 55–74 years who did not have PAD in 1987. After 17 years of follow-up, a total of 208 subjects developed symptomatic PAD. Finally, the levels of various inflammatory parameters in patients with PAD were found to be significantly higher than those in the non-PAD population (30), indicating that PAD is positively correlated with inflammation. In contrast, studies have also shown that AGR is negatively correlated with the level of inflammation (31), indirectly demonstrating the negative relationship between AGR and PAD, which is consistent with the conclusion of this study. In addition, studies have shown that PAD is related to thrombosis (32). AGR is a composite indicator of albumin and globulin, partially reflecting the physiological effects of albumin and globulin. Albumin has antithrombotic properties, and a decrease in albumin level increases blood viscosity, thereby increasing the risk of thrombosis (11). Specifically, albumin inhibits platelet aggregation by binding to and neutralizing the effects of thromboxane A2 and enhances antithrombin III activity, thereby reducing fibrin formation (33, 34). Especially in people with hyperproteinemia, the risk of thrombosis is higher than that of the natural population. Conversely, an increase in globulin can also lead to increased blood viscosity compared with albumin, thereby increasing the risk of thrombosis (12). Therefore, considering the relationship between albumin and globulin and thrombosis, a decrease in AGR may also result in an elevated risk of PAD by increasing the risk of thrombosis, which is also consistent with the results of this study.

The subgroup analysis revealed that the association between the AGR and peripheral arteries was more pronounced in the smoking population. A previous study analyzed 13, 355 people aged 45–64 years who did not have PAD, coronary heart disease, or stroke. Over a median follow-up time of 26 years, a total of 492 people were diagnosed with PAD. The study found that the number of smoking years was correlated with PAD, showing a stronger correlation than that of stroke and coronary heart disease (35). Furthermore, smoking increases the risk of PAD (36); the underlying pathological mechanism can be briefly described as follows: 1. Smoking is associated with a variety of inflammatory factors, which can accelerate the process of atherosclerosis and lead to local stenosis; 2. Smoking causes endothelial cell dysfunction, inhibiting the effective contraction and relaxation of vascular smooth muscle, resulting in functional ischemia; 3. Smoking can also increase blood coagulability and platelet reactivity, and increase the risk of thrombosis (37–39); moreover, a decrease in AGR can also promote inflammation, atherosclerosis formation, and thrombosis risk.

The association between AGR and PAD showed a borderline significant interaction in the stroke population. According to statistics, the risk of recurrent stroke in stroke patients is higher than that in non-stroke patients, and the risk of PAD in stroke patients is also significantly higher than that in the natural population (40). Previous studies have shown higher inflammation levels in the stroke population, accompanied by enhanced platelet activity, leading to an elevated risk of thrombosis and recurrent stroke (41). Moreover, stroke patients are more likely to have additional risk factors for thrombosis, facilitated by a decrease in AGR.

Nevertheless, the limitations of this study should be acknowledged. First, the cross-sectional study design cannot prove the causal relationship between the AGR and peripheral arteries. Second, although we excluded individuals with known malignancies and severe liver dysfunction, other conditions that profoundly affect AGR, such as massive proteinuria, multiple myeloma, and rheumatic immune diseases, could not be systematically excluded due to the unavailability of these specific data in the registry. This may introduce residual confounding. Third, although many covariates have been adjusted for, residual confounding cannot be completely excluded. Furthermore, the study did not collect data on dietary patterns, such as plant-based diet indices or the inflammatory potential of the diet, which are known to influence both serum albumin levels and vascular health, potentially contributing to unmeasured confounding.

Still, this study benefits from multiple strengths. First, this study is a multicenter large-cohort study. Second, to the best of our knowledge, this study is the first to explore the relationship between AGR and PAD in the hypertensive population. Third, the association between the AGR and PAD is more pronounced in the smoking and stroke populations, adding further clinical relevance to the findings.

## Conclusion

This retrospective large-cohort cross-sectional study revealed an inverse association between AGR and PAD, which is more pronounced in smokers and stroke patients. Although this study has found an inverse association between the AGR and PAD, little relevant evidence is available. In the future, longitudinal cohorts and artificial control of the AGR are needed to further evaluate the risk of PAD. Overall, AGR is an easily accessible indicator in clinical practice and may become a new risk stratification indicator or a marker of improved risk assessment for PAD in the future.

## Data Availability

The datasets presented in this study can be found in online repositories. The names of the repository/repositories and accession number(s) can be found in the article/**Supplementary Material**.
