# Supplementary material for: The role of albumin–globulin ratio in peripheral arterial disease among hypertensive adults: evidence from a large-scale multicenter study
Source: Front Endocrinol (Lausanne). 2026 May 28;17:1839770. doi: 10.3389/fendo.2026.1839770 (PMC13253312; doi:10.3389/fendo.2026.1839770)
Supplement: Supplementary file 1 [file DataSheet1.docx]

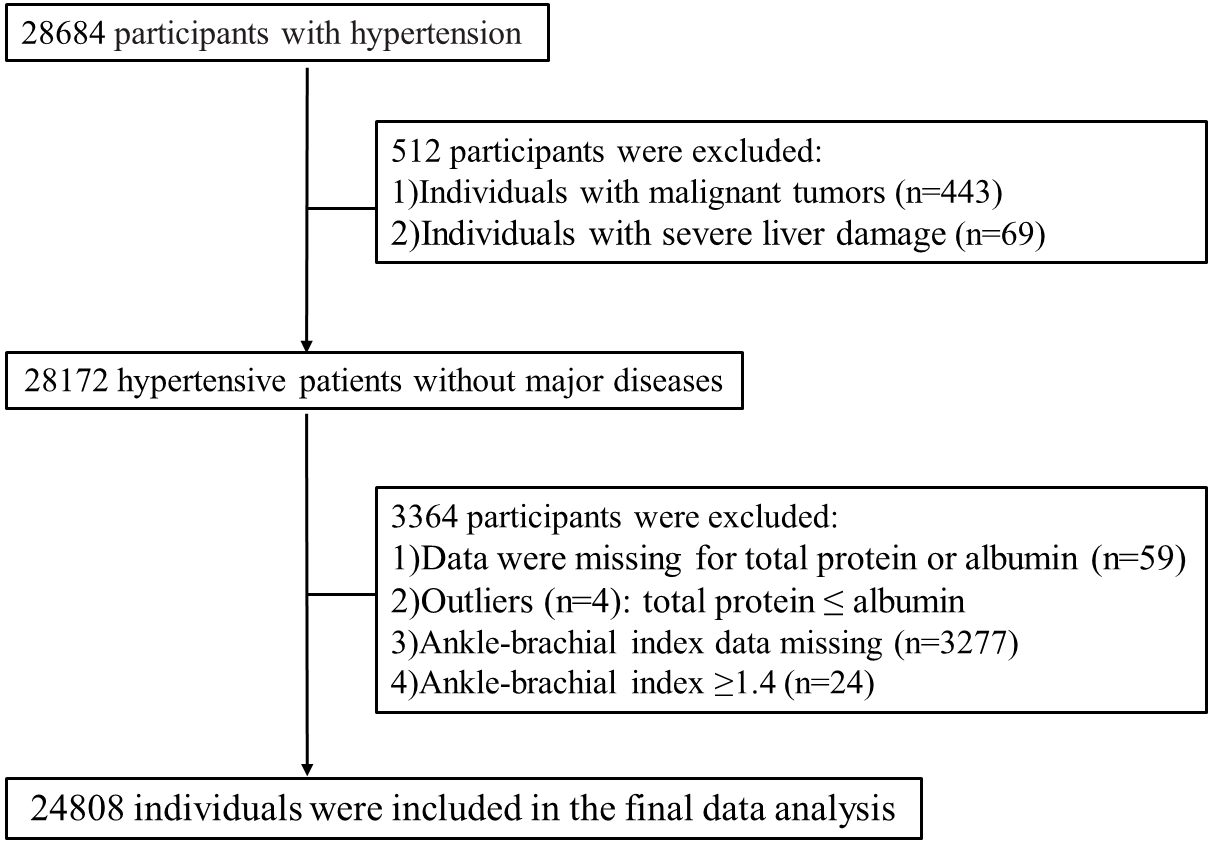


**Figure S1.** Flow diagram of the study participants.

**Table S1. Post-hoc sensitivity analysis of the association between AGR (Q1–Q2 combined as reference) with PAD in hypertensive adults**

| **Variables** | **Events / N** | **PAD OR (95% CI), *p* value** | | | |
| --- | --- | --- | --- | --- | --- |
|  |  | **Crude model** | **Model 1** | **Model 2** | **Model 3** |
| AGR Categories |  |  |  |  |  |
| Q1-2 (<1.70) | 361 / 12395 | Ref. | Ref. | Ref. | Ref. |
| Q3 (1.70, <1.86) | 126 / 6203 | 0.69 (0.56, 0.85), <0.001 | 0.73 (0.59, 0.89), 0.003 | 0.78 (0.63, 0.96), 0.021 | 0.77 (0.63, 0.95), 0.017 |
| Q4 (≥1.86) | 108 / 6210 | 0.59 (0.47, 0.73), <0.001 | 0.63 (0.50, 0.79), <0.001 | 0.71 (0.56, 0.89), 0.003 | 0.69 (0.55, 0.87), 0.002 |
| *P* for trend |  | <0.001 | <0.001 | 0.001 | <0.001 |

Model 1 was adjusted for sex, age, BMI, current smoking and current alcohol drinking

Model 2 was adjusted for Model 1 plus SBP, DBP, homocysteine, fasting serum glucose, LDL-C, HDL-C, eGFR, diabetes, stroke, coronary heart disease, antihypertensive drugs, glucose-lowering drugs, lipid-lowering drugs and antiplatelet drugs.

Model 3 (fully adjusted) was adjusted for Model 2 plus AST and ALT.

Abbreviations: PAD, peripheral arterial disease; OR, odds ratio; 95% CI, 95% confidence interval; AGR, albumin globulin ratio; BMI, body mass index (Calculated as weight in kilograms divided by height in meters squared); SBP, systolic blood pressure; DBP, diastolic blood pressure; AST, Aspartate Aminotransferase; ALT, alanine aminotransferase; HDL-C, high-density lipoprotein cholesterol; LDL-C, low-density lipoprotein cholesterol; eGFR, estimated glomerular filtration rate.
